# Evolution of protein interfaces in multimers and fibrils

**DOI:** 10.1063/1.5086042

**Published:** 2019-06-12

**Authors:** W. Jeffrey Zabel, Kyle P. Hagner, Benjamin J. Livesey, Joseph A. Marsh, Sima Setayeshgar, Michael Lynch, Paul G. Higgs

**Affiliations:** 1 Department of Physics and Astronomy, McMaster University, Hamilton, Ontario L8S 4M1, Canada; 2 Department of Physics, Indiana University, Bloomington, Indiana 47405, USA; 3 MRC Human Genetics Unit, Institute of Genetics and Molecular Medicine, University of Edinburgh, Edinburgh EH4 2XU, United Kingdom; 4 Biodesign Center for Mechanisms of Evolution, Arizona State University, Tempe, Arizona 85287, USA

## Abstract

A majority of cellular proteins function as part of multimeric complexes of two or more subunits. Multimer formation requires interactions between protein surfaces that lead to closed structures, such as dimers and tetramers. If proteins interact in an open-ended way, uncontrolled growth of fibrils can occur, which is likely to be detrimental in most cases. We present a statistical physics model that allows aggregation of proteins as either closed dimers or open fibrils of all lengths. We use pairwise amino-acid contact energies to calculate the energies of interacting protein surfaces. The probabilities of all possible aggregate configurations can be calculated for any given sequence of surface amino acids. We link the statistical physics model to a population genetics model that describes the evolution of the surface residues. When proteins evolve neutrally, without selection for or against multimer formation, we find that a majority of proteins remain as monomers at moderate concentrations, but strong dimer-forming or fibril-forming sequences are also possible. If selection is applied in favor of dimers or in favor of fibrils, then it is easy to select either dimer-forming or fibril-forming sequences. It is also possible to select for oriented fibrils with protein subunits all aligned in the same direction. We measure the propensities of amino acids to occur at interfaces relative to noninteracting surfaces and show that the propensities in our model are strongly correlated with those that have been measured in real protein structures. We also show that there are significant differences between amino acid frequencies at isologous and heterologous interfaces in our model, and we observe that similar effects occur in real protein structures.

## I. INTRODUCTION

Many features of cellular biology are governed by the actions and interactions of proteins, and an understanding of their evolution is crucial to understand the evolution of life itself. An important property of proteins is the formation of complexes consisting of two or more subunits, with 30%–50% forming homo-oligomers composed of identical monomers.^1^ Homodimers constitute the majority (41%) of oligomeric proteins of known structure.^2^ Two identical proteins can aggregate in a closed way, with isologous (i.e., head-to-head) interfaces, or in an open way, with heterologous (i.e., head-to-tail) interfaces. If open, they have the possibility of forming infinite fibrils. Amyloid fibrils, formed by normally soluble proteins that assemble to form open insoluble fibers, are resistant to degradation, and their formation can accompany a variety of human diseases, including Alzheimer’s disease, type-2 diabetes, and spongiform encephalopathies.^3^ Given the importance of homo-oligomers in the cellular repertoire, from mediating gene expression to functioning as enzymes, ion channels, and receptors,^4^ it is important to understand the competition between these different ways of assembling. More generally, mutations of amino acids at protein-protein interfaces are known to have large effects on human health because they affect the formation of protein complexes.^5^

Previous theoretical works have modeled protein fibrillogenesis based on mass action kinetics^6^ and thermodynamics of peptide solutions including formation of protofilament intermediates.^7,8^ In this work, we present a simple model that allows both the physical and the evolutionary aspects of protein aggregation to be addressed. Our approach is similar to other previous works^9,10^ in adopting a transfer matrix approach to obtain the equilibrium concentrations of oligomers of different lengths as a function of the free energies of interaction between proteins.

The novelty of our work is that it connects the statistical physics of protein aggregation to the evolution of higher-order protein structure by using population genetics theory to calculate the expected frequency of each protein in the ensemble of sequences generated by mutation and natural selection. We consider cases where the fitness is independent of whether the protein aggregates and cases where fitness is a function of structure, including selection for the formation of dimers and selection both for and against the formation of fibrils.

Our model considers a protein with two possible interacting surfaces, labeled A and B. There are two possible isologous interfaces (AA and BB) and one heterologous interface (AB). The energies of these interfaces depend on the amino acids on the two surfaces, as described in Sec. II. The model allows for the formation of closed dimers, which occur when one or the other of the isologous interfaces is strongly attractive and the other interfaces are weak. It also allows for the formation of fibrils with proteins oriented in the same direction in cases where the heterologous interface is strong or fibrils with proteins aligned in alternating directions in cases where both isologous interfaces are strong. Using the transfer matrix method given in Sec. III, it is possible to calculate the probabilities *P*_*n*_ that a protein is found in an assembly of *n* units. These probabilities depend on the values of the three interface energies.

The multimeric states of proteins are sometimes observed to change rapidly on an evolutionary time scale.^11,12^ This may be an indication of selection for or against multimers or may simply be a result of neutral evolution. Within our model, it is possible to ask how likely dimer and fibril structures are to form under neutral evolution. We include selection in the model using the strong-selection weak-mutation approximation,^13^ which allows the expected frequencies of sequences in the presence of selection to be calculated from their frequencies under neutral evolution. We use a Monte Carlo Markov chain method (Sec. IV) to generate a set of representative protein sequences with frequencies given by evolutionary theory.

Thus, our model provides a simple way of linking evolutionary observations to the underlying statistical physics of protein aggregation. Within this framework, we consider probabilities of formation of dimers and fibrils, both under neutral evolution and under the action of several different kinds of selection. The model also predicts that the frequencies of amino acids at strongly binding interfaces are significantly different from their frequencies under the mutation process alone and from their frequencies at noninteracting, exposed surfaces. Furthermore, the frequencies of amino acids at isologous and heterologous interfaces are found to differ from one another. These predictions are compared with observations of amino acid frequencies at interfaces in databases of real proteins.

## II. CALCULATION OF INTERFACE ENERGIES

**FIG. 1.**
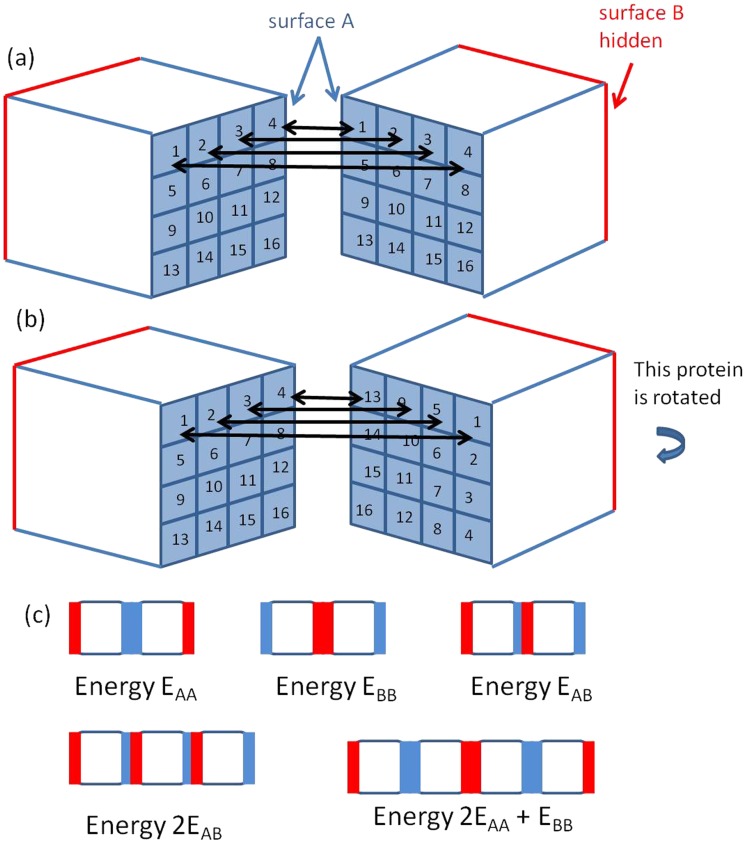
Model of a protein with two opposing surfaces, A and B, which may interact, shown as blue and red, respectively. There are 16 amino acids on each surface. Interface energy is determined by the sum of the energies of the 16 pairwise contacts that are formed when the two surfaces are brought together, as indicated by arrows. (a) An AA interface is shown with the two proteins in the same rotational configuration. (b) An AA interface in which one protein has been rotated by 90°. The energy *E*_*AA*_ of the AA interface is defined as the lowest energy of the four possible rotations. (c) When proteins aggregate in different configurations, the energy of the multimer is given by the sum of the energies of all the interfaces in the multimer structure.

We consider two opposing faces of the protein, denoted A and B, as potential binding surfaces (as shown in Fig. 1). There are two possible isologous interfaces (AA and BB) and one heterologous interface (AB). The energies of the three interfaces *E*_*AA*_, *E*_*BB*_, and *E*_*AB*_ depend on the sequences of residues on the surfaces. Nonsurface residues play no role in this model. A surface is modeled as a 4 × 4 array of amino acids. The energy of an interface is modeled as the sum of the 16 pairwise interactions between amino acids that are formed when two surfaces are brought together (see Fig. 1). We consider four possible 90° rotations of two surfaces. The three energies *E*_*AA*_, *E*_*BB*_, and *E*_*AB*_ are defined to be the lowest of the four energies that arise from the four possible rotations.

The square array of 16 amino acids is used for convenience because we require a simple model for which energies can be calculated for hundreds of thousands of protein sequences during evolutionary simulations. However, the fact that we consider the lowest energy of the multiple rotations of the two surfaces is an important feature of the model. When two identical proteins form an isologous interface, each pairwise interaction between the two surfaces is present twice. This means that the variance of the energy of the interface is twice what it would be for an interface between two independent proteins with the same number of amino acid contacts. The relevant interaction energy controlling binding of two proteins is the lowest energy of the rotational configurations possible when they are brought into contact. As the distribution of energies is broader for homodimers than heterodimers, the lowest energy tends to be lower.^14,15^ This contributes to the excess of interactions between identical proteins and between closely related paralogs that are observed in the analysis of protein-protein interaction networks.^16,17^ This factor is relevant here because we wish to consider relative probabilities of aggregation of multimer proteins in configurations that can involve either isologous or heterologous interfaces. If we simplified our model further by allowing only one rotational configuration, we would lose this effect.

As we wish to distinguish strongly and weakly interacting surfaces, it is useful to define the A surface such that the AA interface is stronger than the BB interface, i.e., *E*_*AA*_ ≤ *E*_*BB*_ (negative energies denote favorable interactions). For each amino acid sequence considered, we simply relabel the A and B surfaces if necessary so that this condition is true.

We use a simple model of pairwise contact energies because we wish to study evolution of large numbers of protein sequences using a model where the fitness depends on the energies of the surface interactions (as described in Sec. III). Thus, it is necessary to be able to evaluate the surface energies of any given sequence very rapidly, which could not be done if a more realistic, three dimensional model of a protein surface was used (for example, as in Refs. 18–20). Although pairwise amino acid potentials leave out many details (e.g., water and ion-mediated interactions, local flexibility of proteins, and the atomic structure of each residue), they have proved to be useful in many ways. The frequently cited early work of Miyazawa and Jernigan^21^ used the frequencies of contacts between amino acid pairs in globular protein structures to construct an effective pair potential matrix. This matrix has continued to be used in many applications such as coarse-grained simulations of protein complexes^22,23^ and is also used as a basis of recent structural models of rates of amino acid substitutions.^24–26^

Interactions between a solvent and amino acids were not included originally,^21^ but Betancourt and Thirumalai^27^ showed that this could be accounted for by shifting the elements relative to the amino acid threonine. The original matrix would not be suitable for our study here because all the energies are negative, meaning all random surfaces would be attractive. This is not true in the transformed matrix, which has both positive and negative elements. The transformed matrix, *B*_*ij*_, is shown in Table I of the supplementary material. It captures the fact that the interactions between pairs of hydrophobic amino acids are substantially negative and those between hydrophobic and polar or between two polar residues are on-average weaker and can be either positive or negative. It also captures specific features such as attractions and repulsions between charged amino acids. As a concrete example, this matrix has been successfully used in a study of protein folding in the GroEL cavity.^28^

It should also be noted that the *B*_*ij*_ matrix we use is derived from contact frequencies within globular protein structures, not from specific frequencies of amino acids at surfaces and interfaces. It is therefore essentially independent of data on interface propensities. We will show here that use of this energy matrix in our model leads to useful predictions on interface propensities that correlate with experimental observations. These predictions are noncircular, whereas they would be if we had used statistical potentials derived from surface data.

## III. CALCULATION OF AGGREGATION PROBABILITIES

For any given sequence of surface residues, we calculate the interface energies as in Sec. II. We then use the interface energies to calculate the probabilities of protein-protein interactions. We consider a solution of a single kind of protein with total concentration *ϕ* moles per unit volume. We determine the equilibrium concentration of monomers *c* and of aggregates of *n* subunits, *C*_*n*_, in the following way.

For each of the three types of interface *ij* ∈ {*AA*, *AB*, *BB*}, we define

$$a_{ij}=\frac{1}{\omega }e^{-\beta E_{ij}},$$ (1)

where *ω* is the number of possible orientational configurations of one protein relative to its neighbor. For the simple cubic lattice considered here, *ω* = 24, which is the number of possible orientations of a cubic object on a cubic lattice. In the calculations below, the statistical weight of an interface of type *ij* is given by *a*_*ij*_*c*/*c*_0_, where *c*_0_ = 1M is the reference concentration.

The concentrations of the different possible aggregates can be calculated by considering formation of chains that grow from one end only. If chains grow from both ends or if chains can aggregate with other chains (rather than just chains with monomers), this does not alter the equilibrium frequencies of the different aggregates. Therefore, we give the simplest case of the calculation here, which allows for the growth of one monomer at a time from one end only.

Letting *C*_*n*_(*A*) and *C*_*n*_(*B*) denote the equilibrium concentrations of a chain of length *n*, with the A or the B face exposed at the growing end, the equilibrium concentrations can be calculated using a transfer matrix method,

$$\left(\begin{array}{c} C_{n}\left(A\right)\\ C_{n}\left(B\right) \end{array}\right)=\left(c/c_{0}\right)\left(\begin{array}{cc} a_{AB} & a_{BB}\\ a_{AA} & a_{AB} \end{array}\right)\left(\begin{array}{c} C_{n-1}\left(A\right)\\ C_{n-1}\left(B\right) \end{array}\right).$$ (2)

We define *C*_1_(*A*) = *C*_1_(*B*) = *c*/2 so that the sum of the two orientations is equal to the total free monomer concentration, *c*. The eigenvalues of the transfer matrix, ***a***, are given by $\lambda_{\pm }=a_{AB}\pm \sqrt{a_{AA}a_{BB}}$, and from these, the abundance of chains of length *n*, given by *C*_*n*_ = *C*_*n*_(*A*) + *C*_*n*_(*B*), can be obtained as

$$\frac{C_{n}}{c_{0}}={\left(\frac{c}{c_{0}}\right)}^{n}\left[A_{+}\lambda_{+}^{n-1}-A_{-}\lambda_{-}^{n-1}\right],$$ (3)

where

$$A_{\pm }=\frac{a_{AA}+a_{BB}\pm 2\sqrt{a_{AA}a_{BB}}}{4\sqrt{a_{AA}a_{BB}}}.$$ (4)

For example, it is easily verified from Eq. (3) that for *n* = 2, the dimer concentration is

$$\frac{C_{2}}{c_{0}}=\frac{1}{2}{\left(\frac{c}{c_{0}}\right)}^{2}\left(a_{AA}+2a_{AB}+a_{BB}\right),$$ (5)

where the terms for the two dimer configurations with isologous interfaces and the two orientations of the dimer with the heterologous interface can be clearly seen.

The concentration of monomers, *c*, can now be determined. The concentration of proteins in clusters of size *n* is given by *ϕ*_*n*_ = *nC*_*n*_. The total protein subunit concentration is *ϕ* = *∑*_*n*_*ϕ*_*n*_. This sum gives an equation from which the free monomer concentration, *c*, can be calculated,

$$\frac{\phi }{c_{0}}=\frac{A_{+}\left(c/c_{0}\right)}{{\left(1-\lambda_{+}\;c/c_{0}\right)}^{2}}-\frac{A_{-}\left(c/c_{0}\right)}{{\left(1-\lambda_{-}\;c/c_{0}\right)}^{2}}.$$ (6)

There is always a single solution to Eq. (6) in the physical range where 0 < *c* < *ϕ*.

The probability of a subunit being present in an *n*-mer is *P*_*n*_ = *ϕ*_*n*_/*ϕ*. The fractions of proteins present as monomers and dimers are *P*_1_ and *P*_2_. We refer to all aggregates of 3 or more units as fibrils; hence, the fraction of proteins in fibrils is *P*_*fib*_ = *∑*_*n*≥3_*P*_*n*_. In some cases, we wish to distinguish closed dimers with the strong AA interface from other dimers. The fraction of proteins in closed dimers is

$$P_{2}^{*}=P_{2}\frac{a_{AA}}{a_{AA}+a_{BB}+2a_{AB}}.$$ (7)

Likewise, in other cases, we wish to distinguish oriented fibrils containing only AB interfaces from general fibrils containing mixtures of all three types of interface. The concentration of proteins in oriented fibrils of length *n* is

$$\phi_{n}^{ori}=\frac{nc^{n}a_{AB}^{\left(n-1\right)}}{{c_{0}}^{\left(n-1\right)}},$$ (8)

and the fraction of proteins in oriented fibrils is

$$P_{ori}=\frac{1}{\phi }\underset{n\geq 3}{\sum }\phi_{n}^{ori}.$$ (9)

## IV. EVOLUTIONARY COMPUTATIONS

We now consider the evolution of proteins whose interactions are described by the statistical physics model above. We consider a population of individuals, each with a gene for the protein in question. The fitness of an individual is a function of the protein sequence. If mutation is weak in comparison to selection, as we will assume below, there is a dominant variant of the protein in the population at any one time, and occasionally, a new variant spreads through the population and replaces the old one. We would like to calculate the long-term steady state frequencies of sequences in the ensemble of sequences generated by this evolutionary process.

We consider protein sequences evolving under a mutational model in which the rate of mutation from amino acid *i* to *j* is *r*_*ij*_ = *uπ*_*j*_, where *u* is a rate constant and *π*_*j*_ is the steady state frequency of amino acid *j* under the mutational process. For simplicity, we deal with mutations at the level of the protein sequence and do not consider the underlying DNA. In the neutral case, protein sequences evolve under the influence of mutation, and there is no selection. Let $f_{k}^{mut}$ be the steady state frequency of sequence *k* under mutation. We consider the simplest case where all 20 amino acids have equal frequency (*π*_*j*_ = 0.05 for all *j*). Hence, there are 20^32^ possible amino acid sequences, each with steady state frequency $f_{k}^{mut}={\left(0.05\right)}^{32}$.

We define the fitness of a sequence as w = 1 + *s*, where positive and negative values of the selection coefficient, *s*, denote advantageous or deleterious sequences and *s* = 0 for neutral variants. For any amino acid sequence, we assume that *s* is a function of the multimer configuration probabilities *P*_*n*_ for that sequence. We consider several choices of fitness functions: (i) a neutral case, where *s* = 0 for every sequence; (ii) positive selection in favor of dimer formation, where $s=\sigma P_{2}^{*}$; (iii) selection against fibril formation, where *s* = −*σP*_*f ib*_; (iv) selection in favor of fibril formation, where *s* = *σP*_*f ib*_; and (v) selection in favor of oriented fibrils containing only *AB* interfaces, where *s* = *σP*_*ori*_. In all these cases, *σ* is a positive constant that determines the strength of selection.

In order to calculate the steady state frequencies of sequences in the presence of selection as well as mutation, we assume that mutations are rare enough so that only one mutation is segregating at a time in the same gene. This is a common approximation in population genetics that allows analytical progress in a simple way. In this approximation, the stationary frequency of a sequence *k* under the influence of selection is weighted by a factor $e^{2N_{e}s\left(k\right)}$ relative to the case with no selection,^29,30^ where *s*(*k*) is the selection coefficient for this sequence and *N*_*e*_ is the effective population size. The frequency of sequence *k* under selection and mutation is

$$f_{k}^{sel}=\frac{f_{k}^{mut}e^{2N_{e}s\left(k\right)}}{\sum_{j}f_{j}^{mut}e^{2N_{e}s\left(\;j\right)}}.$$ (10)

The practical issue with Eq. (10) is the exponential number of sequences in the sum. It is not possible to exhaustively consider all 20^32^ sequences. We therefore use a Markov Chain Monte Carlo (MCMC) sampling method that generates a large sample of representative protein sequences such that the probability of any sequence arising in the sample is proportional to its steady state frequency. The average properties of the full ensemble are closely approximated by the simple mean of the properties of the sequences in the sample.

The MCMC simulations begin with a random sequence of 32 amino acids. We then generate a descendant sequence via replication with mutation. The probability that an amino acid *i* in the parent is replaced by *j* in the descendant is *r*_*ij*_. The probability that the amino acid remains unchanged is *r*_*ii*_ = 1 − *∑*_*j*_*r*_*ij*_. The value of *u* is not critical as it does not influence steady state frequencies. We found *u* = 0.05 to allow efficient exploration of the sequence space. If there is no selection, then every descendant sequence is accepted into the sample, and the method generates a sample with frequencies proportional to $f_{k}^{mut}$. If selection is acting, we accept or reject the descendant according to its fitness. Let the current sequence be *k*_1_ and the descendant be *k*_2_, and let the selection coefficients for these sequences be *s*(*k*_1_) and *s*(*k*_2_). The difference in fitness between the sequences is Δ*s* = *s*(*k*_2_) − *s*(*k*_1_). To ensure that the frequency of any sequence *k* in the sample is proportional to $f_{k}^{mut}e^{2N_{e}s\left(k\right)}$, as is required, the ratio of acceptance of mutations that increase and decrease fitness must be $e^{2N_{e}\Delta s}$. Our MCMC algorithm does this in the simplest way: it accepts the new sequence with probability 1 if Δ*s* is positive and with probability $e^{2N_{e}\Delta s}$ if Δ*s* is negative. If the new sequence is rejected, a second copy of the old sequence goes into the sample. This method is equivalent to the Metropolis algorithm used for Boltzmann-weighted sampling in physics. We also note that a similar method of evolutionary simulation was used in another model of protein evolution^31^ in which the fitness of a sequence depends on its folding ability and its affinity to another target model.

## V. PHENOTYPE DISTRIBUTIONS

**FIG. 2.**
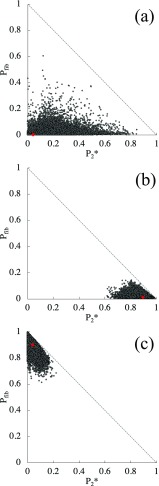
Phenotype distribution in the space $P_{2}^{*}$ vs *P*_*fib*_ for samples of sequences arising under evolution using the MCMC method. (a) Neutral, (b) selection for dimers (*N*_*e*_*σ* = 25), and (c) selection for fibrils (*N*_*e*_*σ* = 25). For each of these plots, the red symbol denotes the mean value of $P_{2}^{*}$ and *P*_*fib*_ for all the sequences in the sample.

The two most useful quantities to summarize the phenotype of a sequence are the frequency of AA dimers, $P_{2}^{*}$, and the frequency of fibrils, *P*_*f ib*_. Figure 2(a) shows the distribution of a sample of sequences generated by the MCMC evolutionary simulation in the neutral case with a total concentration of *ϕ* = 0.01M. The MCMC routine ran for 300 000 generations, and the first 5000 generations were discarded to allow for equilibration. As all sequences have equal frequency under this mutational model when there is no selection, the sequences generated are simply random amino acid sequences. The figure shows that sequences are spread over a broad range of $P_{2}^{*}$ and *P*_*f ib*_. Sequences close to the origin (where $P_{2}^{*}$ and *P*_*f ib*_ are close to zero) exist mostly as monomers (*P*_1_ is close to 1). Sequences in the bottom right corner are mostly dimers. Sequences in the top corner are mostly fibrils. It can be seen, however, that strong fibril formers are rare under neutral evolution at this concentration. Thus, no points are found very close to the top corner in Fig. 2(a). The mean values of these probabilities for all sequences in the sample are $\left\langle P_{2}^{*}\right\rangle =0.04$ and ⟨*P*_*f ib*_⟩ = 0.003. Thus, typical sequences are usually monomers.

Figures 2(b) and 2(c) show the way the phenotype distribution shifts when selection is applied for dimers and for fibrils. When selection is applied for dimers, the distribution shifts close to the bottom right corner, with $\left\langle P_{2}^{*}\right\rangle =0.89$ and ⟨*P*_*f ib*_⟩ = 0.01. When selection is applied for fibrils, the distribution shifts close to the top corner, with $\left\langle P_{2}^{*}\right\rangle =0.04$ and ⟨*P*_*f ib*_⟩ = 0.90. This means that sequences that are either very strong fibril-formers or very strong dimer-formers are possible in this model and that they arise easily when selection favors them. Nevertheless, they are relatively rare compared to the large number of random sequences with weaker interface interactions, so they do not arise frequently in the mixture of random sequences generated under neutral evolution.

**FIG. 3.**
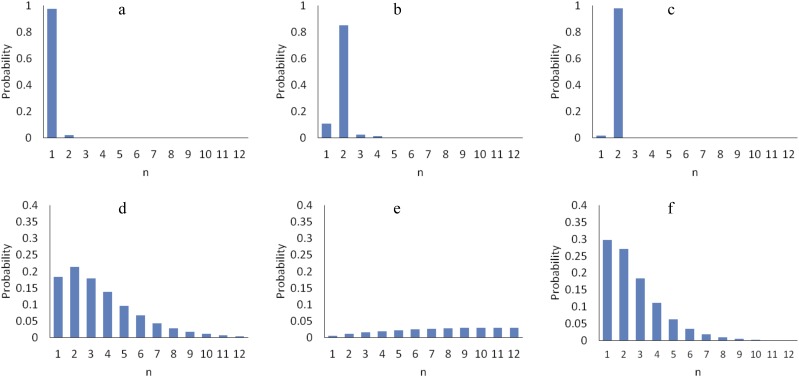
Histograms of *P*_*n*_ for six example sequences illustrating different behaviors at *ϕ* = 0.01M. Panels (a)–(f) refer to the six sequences described in Table I.

To illustrate the range of behaviors shown by individual sequences, we chose the six example sequences A–F described in Table I. For each of these sequences, the distribution of *n*-mer probabilities, *P*_*n*_, is shown in Fig. 3 at concentration *ϕ* = 0.01M. This value is consistent with cellular concentrations of the enzymes that are present at the highest quantities in cells as these are the ones for which aggregation is most relevant. Various mechanisms of subcellular protein localization would additionally enhance their concentrations.^32–34^

**FIG. 4.**
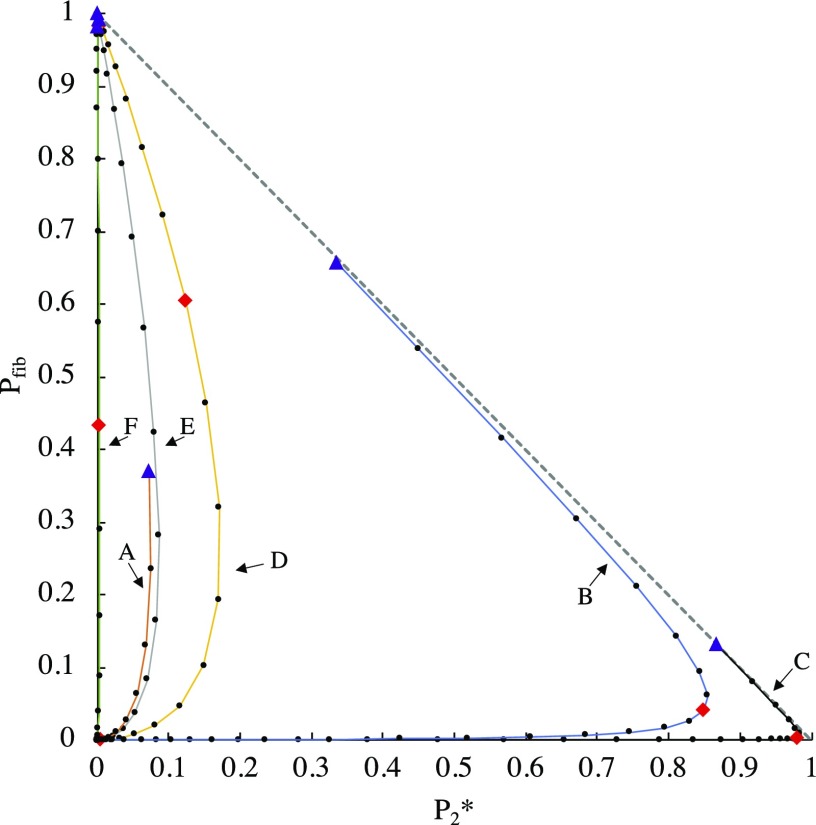
Plot showing trajectories in the space $P_{2}^{*}$ vs *P*_*fib*_ for the six sequences A–F in Table I. The concentration varies from *ϕ* = 10^−6^M to 1M, with the reference concentration *ϕ* = 0.01M labeled as red diamonds and the highest concentration *ϕ* = 1M labeled as purple triangles.

**TABLE I.** Energies of the three interfaces for example sequences A-F discussed in Figs. 3 and 4.

Sequence	Description	*E*_*AA*_/*kT*	*E*_*BB*_/*kT*	*E*_*AB*_/*kT*
A	Monomer	−2.68	−2.32	−2.80
B	Dimer-former	−12.06	−3.14	−5.25
C	Strong dimer-former	−15.82	−0.60	−2.80
D	Fibril former	−9.08	−8.67	−5.68
E	Strong fibril-former	−12.28	−12.06	−12.01
F	Oriented fibril-former	−4.53	−4.52	−8.18

The probabilities *P*_*n*_ change significantly as the concentration is varied. The changes with concentration can be illustrated as trajectories in the $P_{2}^{*}$ vs *P*_*f ib*_ triangle. Figure 4 shows the trajectories for sequences A–F as the concentration is increased from 10^−6^M to 1M. All sequences begin at the origin (all monomers) for low concentration and eventually move toward the fibril corner for very high concentration. Dimer-forming sequences approach the dimer corner at intermediate concentrations. The concentration *ϕ* = 0.01M, which was used in Fig. 3, is shown as red diamonds in Fig. 4. Extreme concentrations higher than this are included in order to illustrate the predictions of the model. The highest concentration point is 1M, shown as purple triangles.

Sequence A is a typical sequence chosen randomly from the sample generated by the neutral simulation [Fig. 2(a)]. The energies of all three interfaces are weak; hence, this sequence is almost entirely monomers at *ϕ* = 0.01M [see Fig. 3(a)]. The trajectory does not move close to the dimer corner at any concentration, and it is still not close to the fibril corner, even at *ϕ* = 1M.

Sequence B is a dimer-former found in the neutral sample. It is the sequence with the highest $P_{2}^{*}$ in Fig. 2(a). This sequence is mostly a dimer at *ϕ* = 0.01M [see Fig. 3(b)] and gradually becomes a fibril at concentrations higher than this. Sequence B forms dimers because the AA interface is strong. *E*_*AA*_ is much lower than the other two energies (see Table I).

Sequence C is a strong dimer-former found in the sample generated under selection for dimer formation [Fig. 2(b)]. It is almost entirely a dimer at the reference concentration and remains very close to the dimer corner even at *ϕ* = 1M. The AA interface is even stronger than for sequence B.

Sequence D is a fibril-former found in the neutral sample. All three interface energies are fairly strong. This sequence has *P*_*f ib*_ = 0.61 at *ϕ* = 0.01M, which is the highest in Fig. 2(a), and the distribution of *P*_*n*_ has significant weight at larger *n*. Sequence E is a strong fibril-former found in the sample of sequences selected for fibril formation [Fig. 2(c)]. All three interface energies are very strong. This sequence has *P*_*f ib*_ close to 1 already at *ϕ* = 0.01M.

Sequence F is a fairly strong fibril-former found in the neutral sample, which has *P*_*f ib*_ = 0.44 at *ϕ* = 0.01M. It differs from the other fibril-formers (D and E) in that the heterologous interface energy *E*_*AB*_ is much lower than the others. This means that it forms mostly oriented fibrils. The frequency of closed AA dimers, $P_{2}^{*}$, is very low at all concentrations; hence, the trajectory in Fig. 4 moves almost along the *P*_*f ib*_ axis.

## VI. PROPERTIES OF PROTEIN INTERFACES

**FIG. 5.**
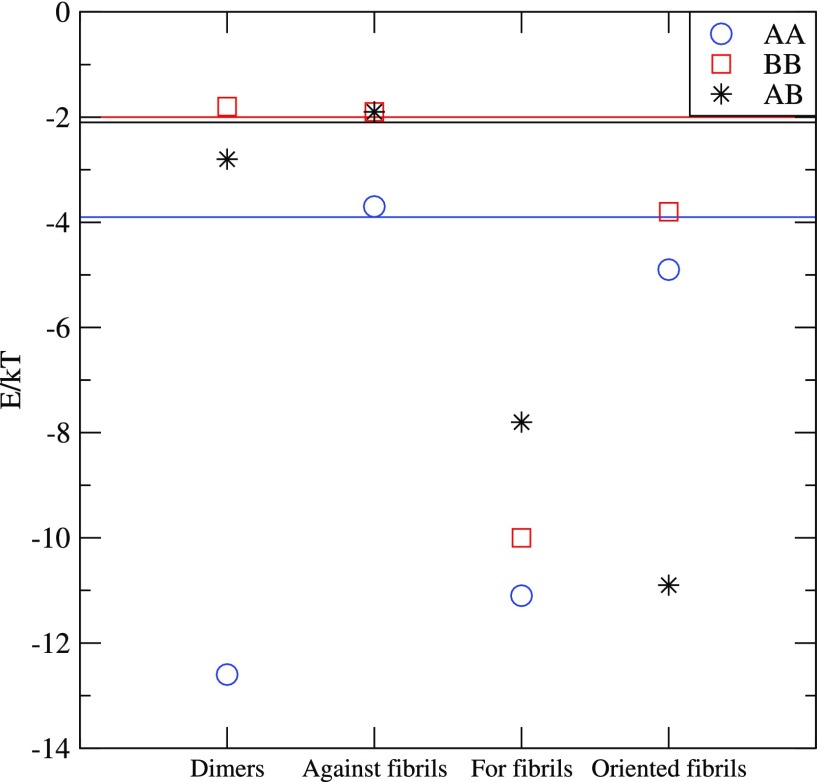
Comparison of the mean energies of the three possible interfaces for random sequences evolving neutrally (shown as horizontal lines) with sequences generated under four different kinds of selection (shown as points). The blue line and circles *E*_*AA*_; the red line and squares *E*_*BB*_; the black line and stars *E*_*AB*_.

Figure 5 shows the mean energies of the three possible interfaces for random sequences evolving neutrally (shown as horizontal lines) and compares these with the mean energies for sequences generated under four different kinds of selection (shown as points). It can be seen that for neutral evolution, *E*_*AA*_ is significantly lower than *E*_*BB*_ even though the sequences are random. This occurs by definition because we have labeled surfaces A and B for each sequence such that A forms the stronger interface of the two. The heterologous interface energy *E*_*AB*_ is intermediate between the two isologous interface energies. All three energies are negative because the mean interaction energy of random amino acid pairs (from the *B*_*ij*_ matrix in Table I of the supplementary material) is slightly negative: ⟨*B*_*ij*_⟩ = −0.057. The mean energy for an interface of 16 random pairs is therefore −0.912. The average energies of the three kinds of interface under neutral evolution are all lower than this because we consider four rotations of the two surfaces, as shown in Fig. 1, and take the lowest of these to define the energy of the interface.

Figure 5 illustrates the way the energies of the interfaces change when selection is applied. In the case of selection for dimers, *E*_*AA*_ decreases substantially with respect to the neutral case, as we would expect, because we are selecting for sequences with high $P_{2}^{*}$. It can be seen that *E*_*BB*_ actually increases slightly with respect to the neutral case. It is important that the BB interface should remain weak because if both AA and BB interfaces become strong, the sequence will form fibrils with proteins in alternating directions.

The second column in Fig. 5 illustrates the case of selection *against* fibrils. We were interested in this case because we expect that uncontrolled fibril formation should be harmful to the cell. Selection against fibrils eliminates the rare sequences with high *P*_*f ib*_ from the neutral phenotype distribution, but since these sequences are rare and since the mean value of *P*_*f ib*_ in the neutral case is already very low, selection against fibrils has only a small effect on the mean energies of the interfaces. It can be seen that all three energies increase slightly with respect to their neutral values, making all kinds of multimers and fibrils less frequent.

There are some proteins whose function requires fibril formation, such as actin and tubulin. Therefore, we also considered the case when selection acts *for* fibrils. In this case, all three interface energies become much lower than the neutral values. It can be seen that *E*_*AA*_ and *E*_*BB*_ decrease more than *E*_*AB*_, meaning that the orientation of proteins in these fibrils will be rather random, but there will be relatively few heterologous interfaces. In Sec. III, we defined *P*_*ori*_, the fraction of proteins in oriented fibrils. For the case where selection is for fibrils of all kinds, we find ⟨*P*_*ori*_⟩ is only 0.05, even though ⟨*P*_*f ib*_⟩, which includes fibrils with proteins in all possible arrangements, is 0.90. By contrast, the fourth case in Fig. 5 shows selection for *oriented* fibrils only. In this case, *E*_*AB*_ decreases much more than *E*_*AA*_ and *E*_*BB*_. Hence, most interfaces will be heterologous. In this case, we find ⟨*P*_*f ib*_⟩ is also 0.90, but ⟨*P*_*ori*_⟩ is 0.87, meaning that almost all the fibrils are oriented.

Taken together, these results show that this model of protein interfaces is quite versatile. It allows selection for both increased and decreased strength of interfaces, and it allows separate selection for either heterologous interfaces (as in the case of dimers) or isologous interfaces (as in the case of oriented fibrils).

## VII. INTERFACE PROPENSITIES OF AMINO ACIDS

Our model allows us to study the way that the frequencies of amino acids at interfaces vary with respect to the frequencies that would be expected under random mutation. The propensities of the amino acids to occur at interfaces have been measured in real proteins (Jones and Thornton^35^ and Levy *et al.*^36^). In this section, we show that our 20-amino acid model generates interface propensities that are similar to these.

**FIG. 6.**
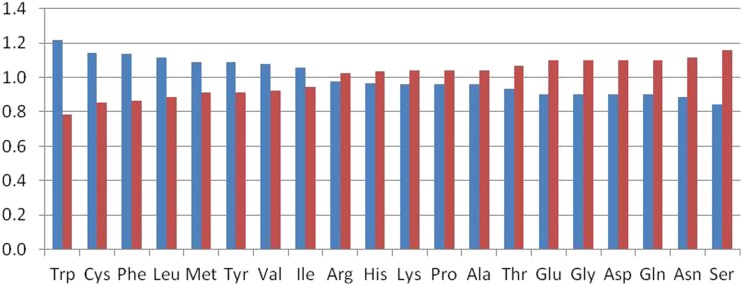
Relative amino acid frequencies at the A surface (*p*_*A*_, blue) and the B surface (*p*_*B*_, red) for neutrally evolving proteins. The frequencies are not equal on the two surfaces because the A and B surfaces have been defined as the stronger and weaker of the two, respectively.

We generated 2 × 10^7^ random sequences of amino acids on the A and B surfaces. We calculated *E*_*AA*_ and *E*_*BB*_, and where necessary, we relabelled the A and B surfaces so that A is the stronger interface. We measured the frequencies *p*_*A*_ and *p*_*B*_ of amino acids on each surface, relative to the expected frequency under neutral mutations, *π*_*i*_ = 0.05. These are shown in Fig. 6, and the data are given in Table II of the supplementary material. When we compare pairs of interfaces in this way and distinguish the stronger from the weaker, the frequencies of amino acids on the two surfaces are different, even though the average frequency on both surfaces has to be equal for all amino acids. Hence, in the figure, we see that *p*_*A*_ can be significantly higher or lower than *p*_*B*_ for many of the amino acids, even though the average of *p*_*A*_ and *p*_*B*_ has to be 1 for every amino acid.

From these probabilities, we define an interface propensity for each amino acid as

$$S_{int}=\ln\left(p_{A}/p_{B}\right).$$ (11)

This score is positive for amino acids that have increased frequency at strong interfaces and negative for those that have decreased frequency.

**FIG. 7.**
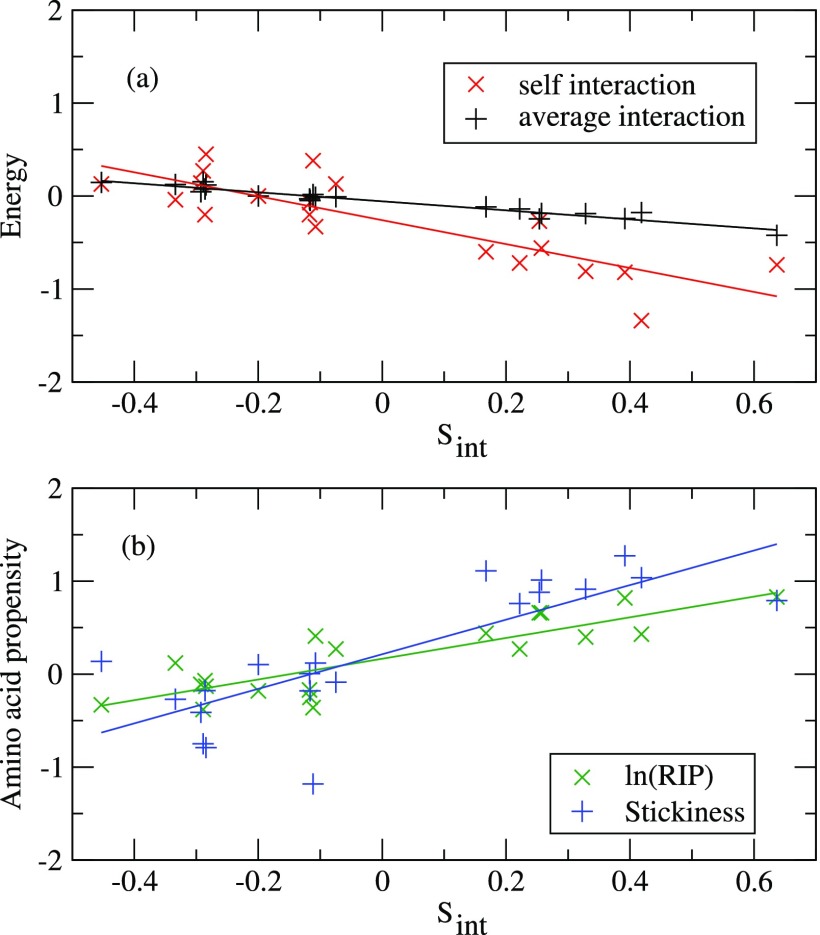
(a) Correlation of *S*_*int*_ with the self-energy *B*_*self*_ and the average energy *B*_*ave*_ of the *B*_*ij*_ matrix. (b) Correlation of *S*_*int*_ with propensities measured in protein data sets. The correlation coefficients and significance values for these plots are *B*_*self*_, *r* = −0.836, *p* = 4.4 × 10^−6^; *B*_*ave*_, *r* = −0.966, *p* = 5.2 × 10^−12^; ln(RIP), *r* = 0.850, *p* = 2.1 × 10^−6^; and stickiness, *r* = 0.797, *p* = 2.6 × 10^−5^.

The *S*_*int*_ scores are related to the energies in the *B*_*ij*_ matrix, as shown in Fig. 7(a). We define *B*_*self*_ as the self-interaction energy of the amino acid (the diagonal element *B*_*ii*_ of the matrix) and *B*_*ave*_ as the mean of the interaction of one amino acid with the 20 possible partners. The more negative the *B*_*self*_ and *B*_*ave*_, the higher the interface propensity. The data are given in Table II of the supplementary material. The correlation coefficients *r* and the *p* values for the *t*-test of correlation are given in the caption and in Table III of the supplementary material. These correlations are highly significant, and the correlation with *B*_*ave*_ is stronger than with *B*_*self*_, as can be seen graphically in Fig. 7(a).

The *S*_*int*_ scores are also related to two previous scales of interface propensities measured from protein structure data. In Table II of the supplementary material, ln(RIP) is the “relative interface propensity” from Ref. 35, and “stickiness” is the interface propensity scale from Ref. 36. Both of these scales are derived from the observed frequencies of amino acids at protein-protein interfaces relative to their frequencies at noninteracting surfaces. The score from our model, obtained from the relative frequencies at the A and B surfaces, is directly comparable to these. Figure 7(b) shows that there is a strong positive correlation between *S*_*int*_ and the two other interface propensities. The correlation coefficients and *t*-test parameters are given in Table III of the supplementary material, showing that there is a highly significant correlation between all three scores.

This observation tells us that the *B*_*ij*_ matrix contains detailed information about the strengths of interactions between the different amino acids that is sufficient to quantitatively predict which amino acids increase or decrease in frequency at interfaces. It also tells us something about why this occurs. Since surface amino acids interact with other copies of themselves and with all possible other amino acids at the interface, those which have the most negative *B*_*self*_ and *B*_*ave*_ increase the most in frequency at the strongly binding interface.

**FIG. 8.**
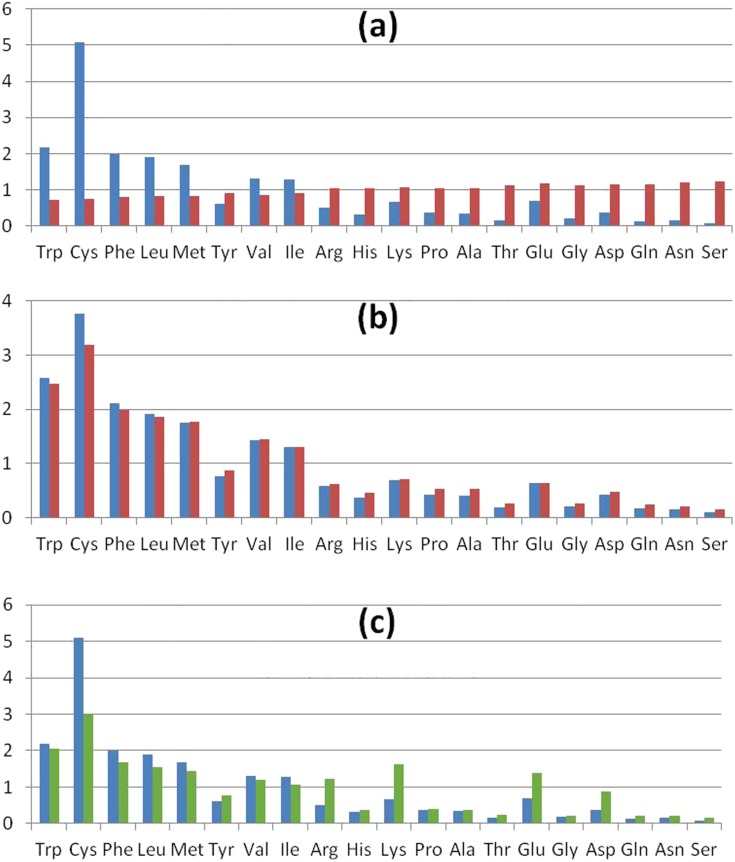
(a) Relative amino acid frequencies at the A surface (*p*_*A*_, blue) and the B surface (*p*_*B*_, red) in the case of selection for dimers. (b) Same in the case of selection for fibrils. (c) Relative amino acid frequencies at the A surface of isologous interfaces in dimers (blue) and the A surface of heterologous interfaces in oriented fibrils (green).

Figure 8 and Table IV of the supplementary material show the frequencies of amino acids at surfaces A and B in the sets of sequences generated by the MCMC sampling method when selection is present. Selection for dimers [as in Fig. 8(a)] accentuates the difference between the A and B surfaces that is already seen in the neutral case (Fig. 6). In the dimer case, the hydrophobic amino acids on the left are very much more frequent on the dimer-forming A interface than on the noninteracting B interface, whereas the hydrophilic amino acids on the right are much more frequent on the noninteracting B interface.

In the case of selection for fibrils, both AA and BB interfaces become strong. Thus, *p*_*A*_ and *p*_*B*_ both show a decreasing trend from left to right in Fig. 8(b), and there is not much difference between *p*_*A*_ and *p*_*B*_. The case of selection *against* fibrils is shown in Table IV of the supplementary material. The frequencies do not change very much from the neutral case because most sequences have a low fibril forming probability, as we saw previously.

Figure 8(c) compares the *p*_*A*_ frequencies in the case of selection for dimers with the *p*_*A*_ frequencies in the case of selection for *oriented* fibrils. In the dimer case, we select for isologous AA interfaces, whereas in the case of oriented fibrils, we select for heterologous AB interfaces. A comparison of these two shows that amino acids differ significantly in frequency between isologous and heterologous interfaces. In particular, it can be seen that Cys is more frequent at isologous interfaces and the charged amino acids (Arg, Lys, Glu, and Asp) are more frequent at heterologous interfaces. From this, we define a propensity for amino acids at isologous vs heterologous interfaces as

$$S_{iso}=\ln\left(p_{A}\left(dimers\right)/p_{A}\left(oriented\mathrm{fi}brils\right)\right).$$ (12)

This propensity is the highest for Cys and the most negative for the charged amino acids (see Table IV of the supplementary material).

**FIG. 9.**
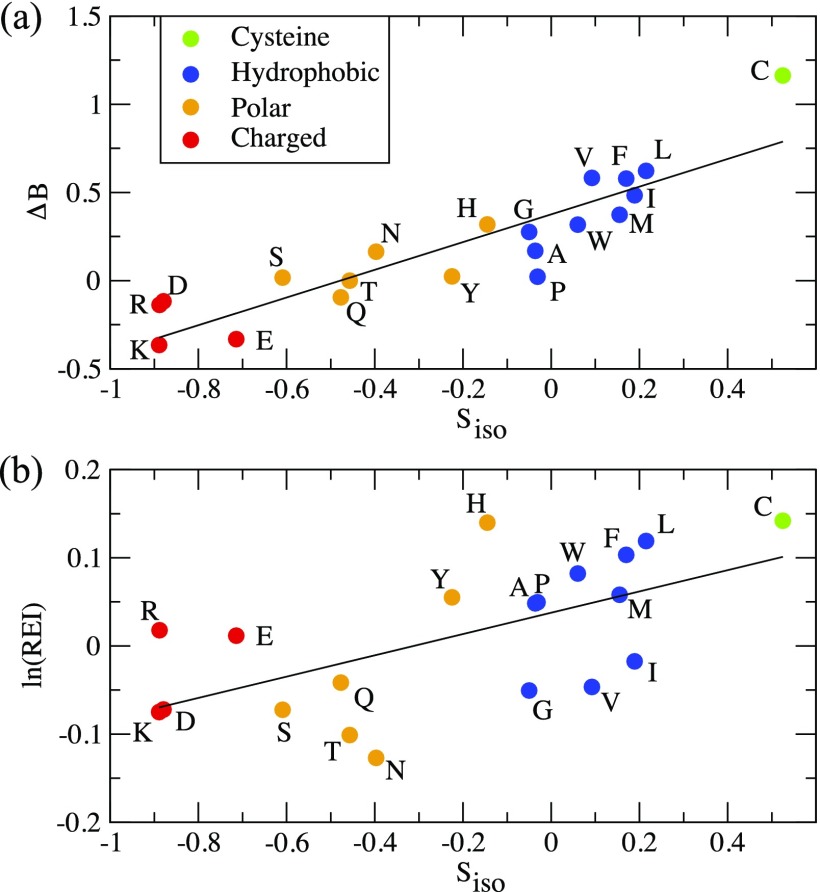
(a) Correlation of the difference between average and self-energies Δ*B* = *B*_*ave*_ − *B*_*self*_ with the isologous vs heterologous interface propensity, *S*_*iso*_. (b) Correlation of the relative enrichment of amino acids in isologous vs heterologous interfaces, ln(REI), found in analysis of real protein complex structures with *S*_*iso*_ obtained from our model. The correlation coefficients and significance values for these plots are Δ*B*, *r* = 0.897, *p* = 8.7 × 10^−8^; and ln(REI), *r* = 0.615, *p* = 3.9 × 10^−3^.

This effect occurs because amino acids in isologous interfaces have a significant probability of interacting with the copy of themselves on the other side of the interface, whereas amino acids in heterologous interfaces only interact with themselves if there is an independently evolved amino acid of the same kind on the other surface. We define the difference between average and self-energies as Δ*B* = *B*_*ave*_ − *B*_*self*_. We expect amino acids with positive Δ*B* to be favored at isologous interfaces, and *vice versa*. Figure 9(a) shows that there is a highly significant correlation of *S*_*iso*_ and Δ*B*. The significance values are given in the caption and in Table III of the supplementary material. From the *B*_*ij*_ matrix in Table I of the supplementary material, it can be seen that Cys has a particularly low value of *B*_*self*_, presumably reflecting the presence of disulphide bridges in the data from which the *B*_*ij*_ matrix was derived. This results in a large positive Δ*B* for Cys. The charged amino acids have positive values of *B*_*self*_, presumably due to repulsions between like charges. This results in negative Δ*B* for the charged amino acids.

It can be seen in Fig. 1(b) that when there is a 90° rotation of one protein with respect to the other, four of the 16 amino acids (numbered 1, 6, 11, and 16) form pairwise contacts with themselves. The same happens in the 270° rotation but does not happen in the 0° and 180° rotations. Although some of these details are particular to the square lattice we are using, the point that amino acids in isologous interfaces can interact with copies of themselves is still true in real proteins where there is no square lattice. For example, the same effect occurs in the circular patch model used in Refs. 14 and 15. Therefore, it is reasonable to ask whether the systematic difference in amino acid frequencies between isologous and heterologous interfaces that we observe in our model also arises in real proteins. To address this, we performed a systematic analysis of the amino acid residues present in the homomeric interfaces of real protein complex structures present in the Protein Data Bank.

Starting from a snapshot of all of the structures in the Protein Data Bank (9-26-2018), all protein residues present in homomeric interfaces were identified as those burying any solvent-accessible surface area with an identical polypeptide chain. Incomplete residues missing any nonhydrogen atoms from the side chain were excluded. Isologous interfaces were classified as those where the correlation between the residue-specific buried surface areas for each subunit in an interacting pair was >0.7, as defined previously.^42^ Since the dataset of interface residues initially contained data from many proteins with closely related or identical sequences, we used the PISCES protein sequence culling server^43^ to remove chains with 90% or greater similarity. This resulted in a total number of 932 536 interface residues from 18 777 nonredundant chains. The total number of occurrences of each amino acid was then counted for isologous and heterologous interfaces, and the proportion was calculated by dividing by the total number of residues at each type of interface. Relative enrichment at isologous interfaces, REI, was calculated by dividing the proportion of each amino acid at isologous interfaces by the proportion at heterologous interfaces (see Table IV of the supplementary material).

Interestingly, we observe a significant correlation between *S*_*iso*_ and ln(REI) [see Table III of the supplementary material and Fig. 9(b)], thus validating the utility of our simplified model and demonstrating its power in capturing genuine sequence differences between the different types of interfaces. The deviations between our model and the pattern observed in real structures could be due to a number of factors. In particular, there are likely to be systematic differences in the functions of homo-oligomers with isologous vs heterologous interfaces as there is a strong association between symmetry and function.^4^ For example, transmembrane channels will be enriched in heterologous interfaces due to their strong association with higher-order cyclic symmetry. Thus, if the interfaces of transmembrane proteins tend to differ in amino acid composition compared to other proteins, this could add a degree of bias.

## VIII. DISCUSSION AND CONCLUSIONS

This work presents a first attempt at a theoretical description of the evolution of multimers and fibrils. The pairwise contact-energy matrix that we used is a simple way of defining interface energies that does not account for the three dimensional structure of surfaces. It was not optimized in any way for the present model. We are therefore very satisfied that several features of the interface propensities and the isologous/heterologous propensities are quite close to those seen in real proteins.

The inevitable presence of hydrophobic residues means that all proteins will be aggregation prone to some extent. Hydrophobic residues in the interior are necessary for proper folding of proteins, and hydrophobic residues on the surface can lead to formation of functional multimeric states. While uncontrolled protein aggregation has been shown to be associated with an increasing number of pathological conditions, including human diseases, due to loss of normal function or gain in toxic activity, fibril formation can also serve functional roles in cases such as adhesion and biofilm formation in bacteria^37^ and defense against micro-organisms.^38^ Cells employ a range of strategies to control aggregation at both the sequence levels (for example, through modulation of aggregation-prone regions or protein stability) and at the cellular level (for example, through compartmentalization and modulation of protein abundance).^39^

With the interaction energies used here, we find that strongly aggregating proteins will be rare under neutral evolution at concentrations that are likely to arise in the cell. This conclusion needs to be treated with caution because of the simplicity of the interaction energy rules. We have only considered solutions of a single kind of protein. It may be possible to extend the model to consider mixtures of many kinds of proteins in the future. It should also be noted that there is a parameter *ω* for rotational entropy in Eq. (1) that is not known with certainty. Lower values of *ω* would lead to higher probabilities of aggregation at any given concentration. Also, we have arbitrarily chosen surfaces with 16 amino acids. Increasing or decreasing the number of interacting amino acids in a patch would increase or decrease the strength of interactions, which would also affect the frequency of strongly aggregating proteins expected under neutral evolution.

A further caveat is that the evolutionary calculations were done under the approximation that a single mutation is segregating in the sequence at once, which is not always true. This could be improved using full-scale population genetics simulations in the future. It would also be possible to consider evolution at the DNA level and determine the protein sequence by translation of the gene. This would allow us to consider cases where the steady state frequencies of the amino acids in the proteins and the four nucleotides in the genes are biased by mutation.

Future extensions of this work include developing this model to consider other multimer structures, such as cyclic and dihedral tetramers, by allowing more than two sticky faces on each protein or by considering proteins with two sticky faces at an angle of 90° to one another. An important aim will be to predict the relative frequencies of multimers of different symmetries and different numbers of subunits, as is tabulated in the “periodic table” classification of the protein structure database.^40^ Furthermore, as our model is able to predict the way the multimer structures will change when mutations are made to the surface residues, we will be able to study evolution of multimer structures over time in a family of related species and compare this with studies of structural evolution.^41^ The present approach therefore introduces a method from which a wide range of new developments will be possible for the study of the evolution of higher order protein structure.

## SUPPLEMENTARY MATERIAL

The supplementary material file Supplementary Tables.xlsx contains the data in the four supplementary tables. The file Supplementary Table Descriptions.docx contains descriptions of these tables.
